# Spread of the *mcr-1* colistin-resistance gene in *Escherichia coli* through plasmid transmission and chromosomal transposition in French goats

**DOI:** 10.3389/fmicb.2022.1023403

**Published:** 2023-01-04

**Authors:** Michaël Treilles, Pierre Châtre, Antoine Drapeau, Jean-Yves Madec, Marisa Haenni

**Affiliations:** ^1^Laboratoire d’Analyse Qualyse, Champdeniers Saint-Denis, France; ^2^Association Régionale de Prévention contre la résistance aux Antimicrobiens, Champdeniers Saint Denis, France; ^3^Unité Antibiorésistance et Virulence Bactériennes, Agence Nationale de Sécurité Sanitaire (ANSES) – Université de Lyon, Lyon, France

**Keywords:** colistin, *mcr-1*, transmission, goat, transposon, IncX4, IncHI2

## Abstract

**Introduction:**

Colistin-resistance widely disseminated in food-producing animals due to decades of colistin use to treat diarrhea. The plasmid-borne *mcr-1* gene has been extensively reported from bovine, swine and chicken worldwide, but smaller productions such as the goat farming sector were much less surveyed.

**Methods:**

We looked for colistin-resistant isolates presenting plasmid-borne genes of the mcr family in both breeding (*n*=80) and fattening farms (*n*=5). Localization of the *mcr-1* gene was performed using Southern blot analysis coupled to short-read and long-read sequencing.

**Results:**

Only the *mcr-1* gene was identified in 10% (8/80) of the breeding farms and four over the five fattening farms. In total, 4.2% (65/1561) of the animals tested in breeding farms and 60.0% (84/140) of those tested in fattening farms presented a *mcr-1*-positive *E. coli*. The *mcr-1* gene was located either on the chromosome (32.2%) or on IncX4 (38.9%) and IncHI2 (26.8%) plasmids. As expected, both clonal expansion and plasmidic transfers were observed in farms where the *mcr-1* gene was carried by plasmids. Tn6330 transposition was observed in the chromosome of diverse *E. coli* sequence types within the same farm.

**Discussion:**

Our results show that the *mcr-1* gene is circulating in goat production and is located either on plasmids or on the chromosome. Evidence of Tn*6330* transposition highlighted the fact that chromosomal insertion does not impair the transmission capability of the *mcr-1* gene. Only strict hygiene and biosecurity procedures in breeding farms, as well as a prudent use of antibiotics in fattening farms, can avoid such complex contamination pathways.

## 1. Introduction

Colistin is an antibiotic compound that has been widely used in animals during decades whereas it has long been disregarded to treat humans – except as last-resort molecule – due to its nephro- and neurotoxicity. Colistin is active against several bacterial species/genus among Enterobacterales (mainly *Escherichia coli*, *Salmonella enterica*, *Klebsiella pneumoniae*, or *Enterobacter* spp.) but also non fermenters, such as *Pseudomonas aeruginosa* or *Acinetobacter baumannii*. Colistin-resistance went globally unnoticed until late 2015, when the plasmid-borne *mcr-1* gene was described in *E. coli* and *K. pneumoniae* isolates from food-producing animals, and also from humans, in China. This discovery raised concerns that colistin-resistance in animals could be transmitted to humans and impair last-line treatments in healthcare settings (Liu et al., 2016). Since then, the number of reports on colistin-resistance due to *mcr* genes grew exponentially, mostly in livestock animals, and new *mcr* genes (to date, up to *mcr-10*) and *mcr* gene variants were identified, albeit with very different epidemiological successes (Valiakos and Kapna, 2021). These variants differ by their degree of homology, their frequency (*mcr-1* is reported globally while *mcr-2* has only been identified a couple of times), their geographical distribution and the bacterial host in which they are circulating (*mcr-1* is widespread in Enterobacterales, while *mcr-4* and *mcr-5* are more restricted to *Salmonella* spp.; Hussein et al., 2021).

The *mcr-1* gene is still by far the most widely disseminated gene among the *mcr* family and has principally been associated with IncI2, IncX4, and IncHI2 plasmids worldwide (Matamoros et al., 2017). The selective advantage of these plasmids may rely on increased fitness, together with high transfer efficiency for IncI2 and IncX4 (Liu et al., 2016; Wu et al., 2018), or on capacity to carry multiple antimicrobial resistance (AMR) genes for IncHI2. Altogether, these three *mcr-1*-carrying plasmids have efficiently disseminated in animals since the 2010s, in livestock as well as in pets and wildlife (Migura-Garcia et al., 2020; Moon et al., 2020; Valiakos and Kapna, 2021), but also in humans and the environment (Anyanwu et al., 2020; Gagliotti et al., 2021). In addition to the widely reported plasmid-borne diffusion, the *mcr-1* gene has also been identified on the chromosome, where it is mostly located within the composite transposon Tn*6330* (Li et al., 2018).

In livestock in France, the *mcr-1* gene has been detected through various AMR surveillance programs in the pig, poultry and bovine sectors (Haenni et al., 2016; Perrin-Guyomard et al., 2016; Delannoy et al., 2017; Haenni et al., 2018; Hamame et al., 2022) where colistin has long been prescribed to treat diseased animals (ANSES, 2020). In 2016, in the frame of the Resapath network (Mader et al., 2021), we also identified the first *mcr-1*-carrying *E. coli* from a goat. Since small ruminants are usually overlooked in AMR surveys despite a strong usage of antibiotics, including colistin in some breeding practices, this finding prompted us to set up a specific study and search for colistin-resistance in breeding and fattening goat farms. The aim of this study was to determine the proportion of *mcr-1-*positive goat farms and to characterize the collected isolates molecularly, first to get better insights into the transmission routes of the colistin-resistant clones and plasmids in this food-producing sector, and second to make the breeders aware of their own breeding/fattening practices in order to improve them.

## 2. Materials and methods

### 2.1. Sampling

Between 2018 and 2019, fecal samples were collected from goats in the Poitou-Charentes area, one of the main goat rearing area in France covering 30% and 70% of the total national goat milk herd and milk production, respectively. Two types of farms were sampled. Each farmer provided written consent for sampling and completed a questionnaire on their husbandry and antibiotic use practices. First, 80 goat breeding farms were visited once. In each farm, rectal swabs from 10 adults and 10 kids (<1 month of age) were collected using e-swabs (Biomerieux, Marcy-l’Etoile, France). In a few cases, the expected number of samples could not be obtained, so that a total of 1,561 animals including 775 kids were sampled. Second, in the same period of time, four fattening farms were visited, corresponding to five fattening batches (two successive batches in the same farm). Over the one-month fattening period, four batches were sampled three times (10 goat kids (1–7 days old) upon arrival, 15 and 30 days after arrival) whereas the fifth batch was sampled only twice (upon arrival and 15 days later) due to unexpected earlier slaughtering.

### 2.2. Bacterial isolation and identification

Samples were sent to the laboratory within 48 h after sampling. A total of 50 μl of the fecal suspension were inoculated in an enrichment broth extemporaneously prepared with 9 ml BHI (Brain Heart Infusion) broth supplemented with one colistin disk (10 μg; Mast Diagnostics, Amiens, France). After an overnight incubation at 35 ± 2°C, 50 μl of the broth were streaked onto a ChromID® Colistin agar (Biomerieux). After 18–24 h incubation at 35 ± 2°C, the identification of one pink colony (suspected to be *E. coli*) was confirmed by MALDI-TOF MS (Bruker, Wissembourg, France). All confirmed *E. coli* were kept at −80°C in peptone water plus glycerol (25%) for further investigations. On all isolates, the presence of the *mcr-1* to *mcr-5* genes was tested using a multiplex PCR (Rebelo et al., 2018), and the presence of the *mcr-9* gene was tested using a simplex PCR (Haenni et al., 2020). *mcr-6* to *mcr-8* and *mcr-10*, which are rarer and more restricted to other Enterobacterales species, were not included in the screening.

### 2.3. Antimicrobial susceptibility testing

Susceptibility testing was performed using disc diffusion on Mueller-Hinton agar (BioRad, Marne-la-Coquette, France), according to the guidelines and clinical breakpoints (human and veterinary) of the Antibiogram Committee of the French Society for Microbiology.^1^ The following discs (Mast Diagnostics) of human and/or veterinary interest were tested: amoxicillin 25 μg, amoxicillin + clavulanic acid 20/10 μg, piperacillin 30 μg, ticarcillin 75 μg, piperacillin + tazobactam 30/6 μg, ticarcillin + clavulanic acid 75/10 μg, cefalotin 30 μg, cefuroxime 30 μg, cefotaxime 30 μg, ceftiofur 30 μg, ceftazidime 30 μg, cefoxitin 30 μg, cefepime 30 μg, cefquinome 30 μg, ertapenem 10 μg, aztreonam 30 μg, streptomycin 10 μg, gentamicin 15 μg, tobramycin 10 μg, netilmicin 30 μg, chloramphenicol 30 μg, florfenicol 30 μg, tetracycline 30 μg, sulfonamides 200 μg, trimethoprim 5 μg, nalidixic acid 30 μg, enrofloxacin 5 μg and ofloxacin 5 μg. Minimum inhibitory concentrations (MICs) were determined by broth microdilution for colistin, according to the European Committee for Antimicrobial Susceptibility Testing (EUCAST). *Escherichia coli* ATCC 25922 was included for quality control both for disc diffusion and broth micro-dilution. All isolates presenting resistance to three or more antibiotic family were considered as Multi-Drug Resistant (MDR; Magiorakos et al., 2012).

### 2.4. Short-read sequencing

DNA was extracted using the NucleoSpin Microbial DNA extraction kit (Macherey-Nagel, Hoerdt, France) according to the manufacturer’s instructions. Library preparation was performed using the Nextera XT technology and sequencing was performed on a NovaSeq-6,000 instrument (Illumina, San Diego, United States). After sequencing, reads were quality trimmed and *de novo* assembled using Shovill v1.0.4 and the quality of assemblies was assessed using QUAST v5.0.2. Quality control statistics of all sequenced isolates are provided as Supplementary Table S1. STs were determined using MLSTFinder v2.0.4 and point mutation in the *pmrAB* genes were identified using PointFinder (CGE online tools, http://www.genomicepidemiology.org/), while resistance genes and replicon content were inferred using ABRicate v1.0.1.^2^ Virulence factors (VFs) were determined using VirulenceFinder and serotypes were determined using SeroType Finder.^3^

### 2.5. Phylogenetic analysis

MLVA characterization was performed prior to sequencing (Caméléna et al., 2019). The cgMLST (core genome multi-locus sequence type) was extracted from the WGS data. The pan-genome was determined, and core gene alignments were generated, for each collection, with Roary v. 3.13.0 (Page et al., 2015) using a Protein BLAST identity of 80% and a core definition of 90%. In the first step, all assemblies were annotated *de novo* with Prokka v1.14.6 using default settings (Seemann, 2014). The Prokka annotations were provided to Roary as input. Subsequently, recombination was removed with gubbins v2.4.1 and a maximum likelihood tree was constructed from the core gene alignment produced by Roary using RAxML v.8.2.12 using default parameters. Pairwise single nucleotide polymorphism (SNP) distances were calculated using snp-dists.^4^ The resulting tree was visualized using iTol v.6^5^ (Letunic and Bork, 2019).

### 2.6. Plasmid characterization

The plasmidic content was determined from the WGS data using PlasmidFinder 2.0.1.^6^ Plasmids carrying the *mcr-1* gene were assigned *in silico* when *mcr-1* was located on the same contig as the plasmidic marker. When *in silico* data showed no co-occurrence on the same contig, plasmids carrying the *mcr-1* gene were detected using S1-PFGE gels (6 V/cm for 20 h with an angle of 120° at 14°C with pulse times ranging from 1 to 30 s) followed by Southern blot using adequate probes for the *mcr-1* gene as well as for the IncX4 and IncHI2 replicons, as previously described (Saidani et al., 2019). When plasmidic location could not be evidenced, the chromosomal location was looked for by PFGE on *I-Ceu*1-digested DNA, followed by Southern blot hybridization using a 16S rDNA probe and probes corresponding to the *mcr-1* gene.

### 2.7. Long-read sequencing

To get better insight into a subset of plasmids, MinION long-read sequencing libraries were prepared according to the Oxford Nanopore Technologies, United Kingdom, using the native barcoding expansion kit (catalog number EXP-NBD104; Oxford Nanopore Technologies) and the ligation sequencing kit (SQK-LSK109). Sequencing was performed on a MinION sequencer using a SpotON Mk 1 R9 version flow cell (FLO-MIN106D; Supplementary Table S1). Assembly of both Illumina short reads and Nanopore long reads was performed using Unicycler (Wick et al., 2017). Annotation was done using RASTtk on the Patric 3.25.0 platform^7^ (Brettin et al., 2015) and the genetic environment of the *mcr-1* gene was explored using BLASTn.

### 2.8. Treatment ratios

The treatment ratios was calculated as the number of treatment administered over 12 months divided by the number of breeding animals in the farm.

### 2.9. Statistics

The Mann Whitney-Wilcoxon test was used to compare the presence of *mcr-1* and non-*mcr-1* colistin-resistant isolates versus the treatment ratio.

### 2.10. Accession number(s)

All genomic sequences were deposited in DDBJ/EMBL/GenBank under the BioProject accession number PRJNA857909.

## 3. Results

### 3.1. Proportion of colistin-resistance and additional resistance genes

**Figure 1:**
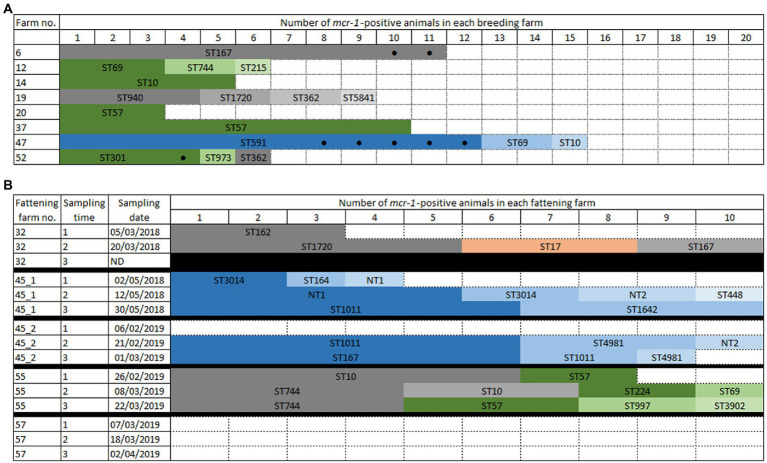
Schematic representation of *mcr-1*-positive *Escherichia coli* in breeding **(A)** or fattening **(B)** farms. The *mcr-1* gene was found on IncX4 (blue colors) or IncHI2 (green colors) plasmids, or on the chromosome (grey colors). **(A)** Each colored box represents a *mcr-1*-positive goat. All boxes correspond to kids, except those marked by a black dot. Empty boxes indicate that no colistin-resistant isolate found. **(B)** In ST17 (orange color), *mcr-1* could not be located. Black boxes indicate that the time point was not sampled. Empty boxes indicate that no colistin-resistant isolate found.

All 80 breeding farms were sampled once (20 animals each) and at least one colistin-resistant (col-R) *E. coli* isolate was identified in 36 of them (36/80, 45.0% of col-R positive farms). Among those 36 col-R positive farms, the *mcr-1* gene was detected in eight of them, leading to an overall 10% (8/80) proportion of *mcr-1*-positive farms (Supplementary Table S2, Figure 1A). The number of *mcr-1*-positive animals per farm varied from 3 to 15 over 20 goats sampled (Figure 1A). In all, over the 1,561 animals tested, 65 (4.2%) were *mcr-1-*positive, of which 57 were goat kids (57/775, 7.4%). *mcr-1*-positive *E. coli* were thus mostly found in kids and, in a given farm, we never found a *mcr-1*-positive adult alone, in the absence of *mcr-1*-positive kids.

In fattening farms, four over the five batches presented *mcr-1* colistin-resistance at least at one sampling time (Figure 1B), whereas no colistin-resistant isolate was found in the last farm (farm #57) at any of the three sampling times. Similarly, no colistin-resistance was observed in farm #45 at the first sampling time of the second batch; however, colistin-resistant *E. coli* were then identified at the second and third time points. Finally, farm #32 was only sampled twice since goats were slaughtered earlier than expected. The number of *mcr-1*-positive animals per time point varied from 3 to 10 over 10 samples, and *mcr-1*-positive *E. coli* were identified in 60.0% (84/140) of the tested animals.

In both breeding and fattening farms, although we looked for the presence of the *mcr-1* to *mcr-5* genes as well as *mcr-9*, no gene or gene variant other than *mcr-1.1* was identified.

### 3.2. Genetic background

**Figure 2:**
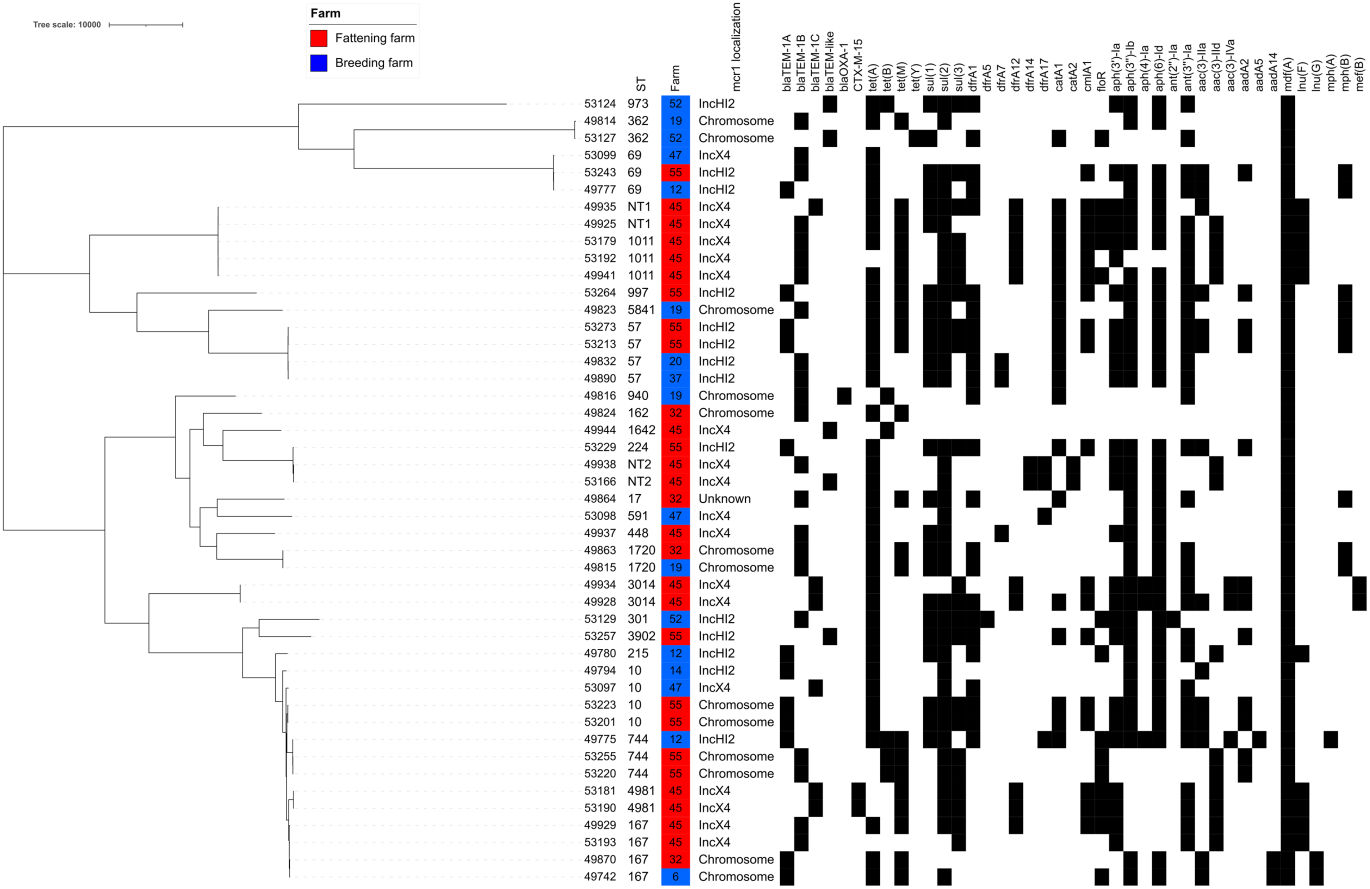
SNP-based phylogenetic tree of all *mcr-1*-positive *E. coli* from goat. The presence of antibiotic-resistance genes is shown by a black square. TEM-like variants are detailed in Supplementary Table S2.

All 149 *mcr-1*-positive isolates were characterized by PCR using the published MLVA scheme (Caméléna et al., 2019), and one representative per profile, per farm and per sampling time was further sequenced. In total, 46 *E. coli* were short-read sequenced, among which 25 different STs were identified (Figure 2; Supplementary Table S2).

In the breeding farms, a total of 13 different STs were found (Figures 1A, 2, Supplementary Table S2). The epidemiological success of identified STs varied within the same farm, with some clones identified only once (for example ST215 in farm #12) and others that had spread locally (for example ST591 – identified in 12 of the 15 *mcr-1* positive isolates from farm #47, or ST167 – identified in all 11 isolates from farm #6). Four STs were found in two different breeding farms (ST10, ST57, ST69, ST362). The virulence genes largely varied depending on the identified clones (Supplementary Table S2), but ST301 has particularly caught our attention. This clone, which was found in four isolates of breeding farm #52, was an O80:H2 enterohemorrhagic EHEC possessing, among others, the *stx2*, *eae* and *ehxA* virulence genes (Cointe et al., 2021).

In the fattening farms a total of 18 different STs were found (Figures 1B, 2; Supplementary Table S2). Of note, NT1 was a single locus variant (SLV) of ST1011, so that the five ST1011 and NT1 isolates only differed by a maximum of 16 SNPs. As two to three sampling campaigns were conducted in each fattening farm, the same ST could be identified over time. When this happened, WGS data proved that this was the same clone in all but one case (2–13 SNPs differences for ST57, ST10, ST744, ST1011, ST3014, non-typable STs NT1 and NT2). The only exception was ST167, which was found in the same farm #45 in 2018 and 2019, but also in two different farms (#32 and #45) 2 months apart. The identified isolates differed, respectively, by 90 and 125 SNPs.

Identical STs were also identified in two to three unrelated breeding or fattening farms on seven other occasions. In five cases (ST10, ST69, ST167, ST362, and ST744), isolates were not genetically related, differing by 29-855 SNPs. In the last two cases, two ST57 (from breeding farm #20 and #37) and two ST1720 (from farm #19 and #32) isolates were identical (0–1 SNP difference). Of note, breeding farm #19 and fattening farm #32 were located on the same geographical site, with specific premises (called breeding farm #19) rearing only adult and kid goats originating from the farm, and other distinct premises (called fattening farm #32) receiving kid goats from farm #19 and other farms of the Poitou-Charentes area.

### 3.3. Genetic localization of the *mcr-1* gene

Based on the sequenced isolates, the *mcr-1* gene was located either on the chromosome (*n* = 48; 48/149, 32.2%) or on the IncX4 (*n* = 58; 38.9%) and IncHI2 (*n* = 40; 26.8%) plasmids. The *mcr-1* gene could not be reliably located on the chromosome or on a plasmid in three ST17 isolates (fattening farm #32; Supplementary Table S2; Figures 1, 2). In most farms, the *mcr-1* gene was identified exclusively on one genetic determinant.

In breeding farms, the *mcr-1* gene was more frequently found on an IncHI2 plasmid (*n* = 5 farms) than on the chromosome (*n* = 3 farms) or on an IncX4 plasmid (*n* = 1 farm). The two plasmids IncX4 and IncHI2 never cohabitated in a same farm, and the *mcr-1* gene was found located either on the chromosome or on an IncHI2 plasmid only in farm #52.

The situation was similar on the fattening farms, with *mcr-1* located on the chromosome in farm #32, exclusively on an IncX4 plasmid in farm #32 (both in 2018 and 2019), and either on the chromosome or on an IncHI2 plasmid in farm #57.

**Figure 3:**
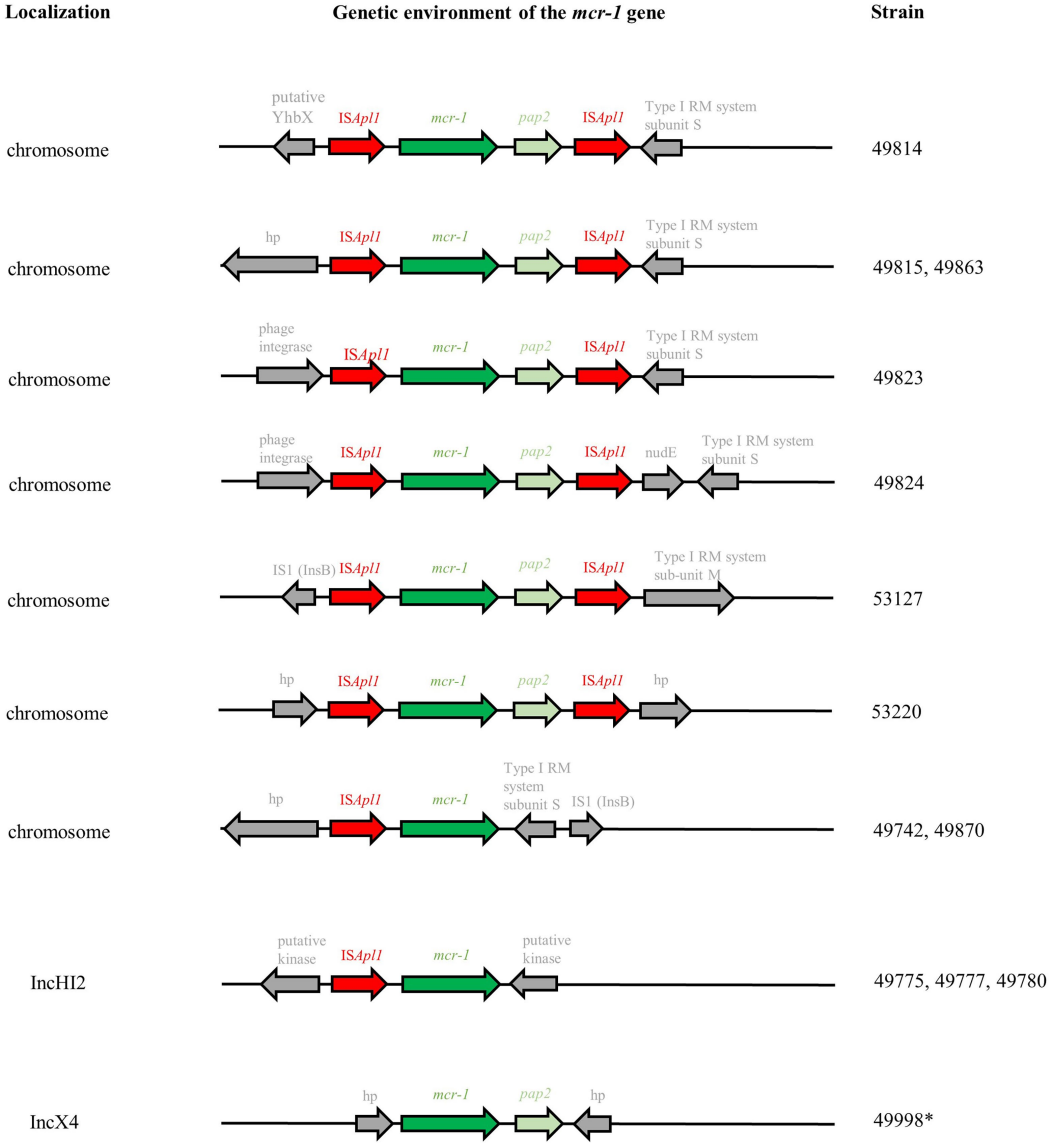
Genetic environment of *mcr-1* genes based on Illumina-ONT hybrid assemblies. RM: restriction-modification; hp: hypothetical protein. *Analyses based on Illumina sequences only.

In four cases (ST167 and ST162 with *mcr-1* located on the chromosome in farms #6 and #32; ST57 with *mcr-1* located on IncHI2 in farms #20 and #37), the *mcr-1* gene had obviously spread in one farm through clonal transmission of one unique clone. On the other hand, plasmid transfer between different *E. coli* isolates was hypothesized for *mcr-1*-bearing IncHI2 plasmids within three farms (#12, #52, #55) and for *mcr-1*-bearing IncX4 within two farms (farm #45, spanning over two batches and five time points, and #47). Long-read sequencing of three isolates (Supplementary Table S2) was thus used to clarify this issue in farm #12, where a *mcr-1*-bearing IncHI2 plasmid was found in three different genetic *E. coli* backgrounds. Sequence analysis showed 99% identity over the 235,257–235,266 bp of the three IncHI2/ST4 plasmids of isolates #49775, #49777, and #49780, strongly arguing for the within-farm spread of a unique *mcr-1*-bearing IncHI2 plasmid between three different *E. coli* isolates. In these IncHI2 plasmids, the *mcr-1* gene alone (without the *pap2* gene) was only preceded by one IS*Apl1* element and inserted in a putative kinase. Illumina sequences showed that, in IncX4 plasmids, the *mcr-1*-*pap2* genes were inserted between hypothetical proteins without any IS*Apl1* element (Figure 3).

Chromosomal insertion of *mcr-1* was also observed in several genetic *E. coli* backgrounds in farms #19, #32, and #55. Long-read sequencing of nine isolates (Supplementary Table S2) showed that the *mcr-1* gene was carried on the prototypic Tn*6330* transposon additionally presenting the *pap2* gene in seven isolates (Figure 3). Tn*6330* was inserted in different locations in the *E. coli* genome, except for isolates #49815 (breeding farm #19) and #49863 (fattening farm #32) which were clonal and presented the Tn*6330* insertion at the identical site. Only isolates #49742 (breeding farm #6) and #49870 (fattening farm #32) displayed a particular and identical genetic arrangement, with the absence of the *pap2* gene and the IS*Apl1* element. Both isolates belonged to ST167, but differed by 54 SNPs.

### 3.4. Additional resistance genes

While all but one sequenced *E. coli* isolate presented genes conferring resistance to aminopenicillins (TEM-like genes), only two of them (from fattening farm #45) presented the Extended-Spectrum Beta-Lactamase (ESBL) *bla*_CTX-M-15_ gene, in coherence with the observed phenotype (Supplementary Table S2). Resistances to tetracyclines (*n* = 45), aminoglycosides (*n* = 43), sulfonamides (*n* = 41), trimethoprim (*n* = 36) and phenicols (*n* = 32) were widespread; the most frequently identified genes were *tet*(A) (*n* = 37), *ant*(3″)-Ia (*n* = 31, conferring resistance to streptomycin/spectinomycin), *sul2* (*n* = 37), *dfrA1* (*n* = 24) and *catA1* (*n* = 19), but several variants of these genes were detected in many isolates (Supplementary Table S2; Figure 2).

In addition to the *mcr-1* gene, seven isolates (7/46, 15.2%) presented mutations in the *pmrA* gene and 33 isolates (71.7%) presented mutations in the *pmrB* gene (Supplementary Table S2). In the *pmrB* gene, the most common mutations were D283G (*n* = 27) and H2R (*n* = 19). Among the mutations that have previously been shown to confer resistance or decreased susceptibility to colistin, the following ones were identified here: G53R and R81S in *pmrA*, each found in one isolate, as well as R93P and P94A in *pmrB*, both found in two isolates.

### 3.5. Antibiotic usage on farms

**Figure 4:**
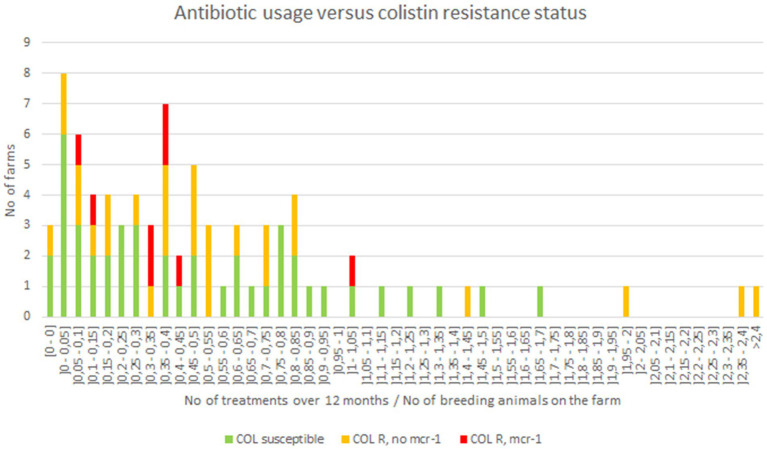
Antibiotic usage in investigated goat breeding farms.

In France, it is of common knowledge that colistin is widely prescribed to 1–7 days old goat kids at arrival in fattening farms, to avoid digestive disorders related to transportation and rearing poorly immunocompetent young animals of mixed origins. Then, animals may also receive antibiotics to treat inter-current diseases over the fattening period. Here, the different treatments and molecules administered per animal in each farm were unfortunately not available. Nonetheless, available data were the treatment ratios per breeding farm, i.e., the number of treatments administered over 12 months divided by the number of adults in the farm (Figure 4). The vast majority of the farms displayed a ratio between 0 (no treatment) to 1 (mean of one treatment per animal over the breeding period; Figure 4). However, when confronting antibiotic exposure ratios in each farm to the proportion of colistin-resistance or *mcr-1*-positive *E. coli* isolates in the same farm, no statistically relevant correlation (*p* > 0.05) was evidenced.

## 4. Discussion

In this study, we investigated to what extend *mcr* genes conferring colistin resistance may have spread within the caprine sector in France. We thus looked for the presence of *mcr-1* to *mcr-5* genes as well as *mcr-9*. The sole *mcr* gene found here was *mcr-1* (*mcr-1.1* variant), which is among the most frequently reported ones in livestock in Europe. In 34/46 isolates, this *mcr-1* gene was accompanied by point mutations in the *pmrA* and *pmrB* genes. Several of these mutations have been reported in other publications including in colistin-susceptible isolates, so that they most probably play no or little role in colistin-resistance (Kuang et al., 2020; Al-Mir et al., 2021). Among the mutation identified here, only a few had previously been associated with colistin-resistance, namely the G53R and R81S mutation in *pmrA* (Sun et al., 2009; Quesada et al., 2015; Rhouma et al., 2016) as well as the R93P and P94A mutations in *pmrB* (Bourrel et al., 2019; Kuang et al., 2020).

The *mcr-1* gene was detected in 10% of the breeding farms, representing 4.2% of all animals and 7.4% of goat kids tested in those farms, respectively. The early treatment of goat (colistin is mostly prescribed to 1–7 days old kids), together with the maturation of the intestinal microbiota, probably explain the fact that resistant bacteria are much more frequently identified in young animals. Nevertheless, this 10% proportion of *mcr-1* positive animals is quite low compared to fattening farms where four over the five tested batches were proved to be *mcr-1*-positive, representing 60.0% of the tested animals. Even if comparison between different contexts and sampling protocols can be hazardous, it is undeniable that the proportion of *mcr-1*-positive *E. coli* is more important in fattening farms than in breeding farms. This difference may reflect different antibiotic selective pressures in the two types of farms, a hypothesis that we were unfortunately not capable to confirm since we had no access to individual prescription records. This hypothesis was also not confirmed by more global antibiotic usage estimates based on number of treatments on breeding farms. However, another important driver of colistin resistance in fattening farms may rely on *mcr-1* gene horizontal spread, which was supported in this study by identical *mcr-1*-positive clones and/or *mcr-1*-bearing plasmids found in different animals of the same farm.

A major feature of the caprine sector also refers to successive steps in the production chain, where breeding farms are the source of male goat kids in fattening farms. Therefore, *mcr-1*-positive animals in a breeding farm can contaminate several fattening farms, where *mcr-1* prevalence may further amplify through close contacts between individuals. The same snowball effect was observed in France for *E. coli* producing ESBLs in the veal calves sector, where ESBL-positive *E. coli* imported from breeding farms were selected by the antibiotic treatment administered to all animals upon arrival on fattening farms (Gay et al., 2019). These ESBL-positive *E. coli* then tended to disappear over the 5–6 months of fattening; a decrease in *mcr-1*-positive *E. coli* was not observed here, either because the goat fattening period may be too short or because the decrease of *mcr-1* positive isolates would not follow the same dynamic as the decrease of ESBL-positive isolates. In any case, as for ESBL-positive isolates, the vast majority of positive animals were kids, and the proportion of resistance dropped drastically with the passage to adulthood. An issue could come from the persistence of *mcr-1*-positive clones in the environment. Indeed, genomic analyses have shown the presence of identical clones (in the case of ST1011 and NT2) 1 year apart, even though the animal batches had been renewed. Also, some STs – such as ST57 or ST1011 – might be particularly prone to carry the *mcr-1* gene. Both STs have recurrently been reported in the poultry production (Maciuca et al., 2019; Dandachi et al., 2020; Al-Mir et al., 2021; Jamin et al., 2021; Sadek et al., 2021) but also in humans (Elnahriry et al., 2016; Al-Mir et al., 2019; Hassan et al., 2021).

Most isolates of this study, which were collected from healthy animals, displayed multiple resistance genes (including third and fourth generation cephalosporins) and virulence factors. This indicates on the one hand that these isolates may be co-selected by the use of different classes of antibiotics, and on the other hand that they may become pathogenic either for the animals or for humans if the context allows. In particular, one adult goat and three kids in breeding farm #52 presented an O80:H2 enterohemorrhagic EHEC possessing the *stx2*, *eae* and *ehxA* virulence genes (Cointe et al., 2021). This serotype was among the three major ones causing pediatric haemolytic uraemic syndrome (HUS) in 2015 in France (Bruyand et al., 2019). Knowing that consumption of raw milk and raw milk cheese is one of the risk factors of EHEC transmission, this finding warrants further surveillance.

In this study, isolates presented the *mcr-1* gene either on the chromosome (32.2%) or on the IncX4 (38.9%) and IncHI2 (26.8%) plasmids. IncI2, which is more widespread in Asian countries was not identified here (Matamoros et al., 2017).

The IncX4 plasmid, even though it was carried by the largest number of *mcr-1*-positive isolates, was found only in one breeding and one fattening farm. This indicates that IncX4 might not be the most widespread vector of *mcr-1*, but suggests that it is highly successful and persistent once established on a farm. Interestingly, the only farm in which *mcr-1* was carried on the IncX4 plasmid (breeding farm #47) was selling goat kids to the only fattening farm (#45) in which *mcr-1* was also circulating on the IncX4 plasmids. Even though we did not identify one identical ST between these two farms, the hypothesis of an IncX4/*mcr-1* plasmid spread is very likely since this plasmid was found in numerous different genetic backgrounds, showing its high transfer ability. In this farm #45, the first sampling point presented either few (in 2018) or no (in 2019) *mcr-1*-positive *E. coli*, while 90–100% of the sampled animals were positive at the second and third time points, probably indicating an on-farm dynamic of *mcr-1* acquisition over time. We also observed the persistence of the ST1011 *E. coli* over 1 year.

The *mcr-1* gene was carried by the IncHI2 plasmid in five out the eight farms sampled, and in one of the fattening farms (#55). We observed both clonal diffusion and plasmidic transfers. The ST57 was found in two breeding farms (#20 and #37) that apparently share no epidemiological link, and at two time points of the fattening farm #55 which did not receive goat kids from farms #20 and #37. The common source for these three farms, if any, remains unknown. Long-read sequencing proved plasmidic transfer in farm #12 between the ST69, ST744 and ST215 genetic backgrounds. The plasmid was an IncHI2 plasmid of ~235kbp additionally carrying the *bla*_TEM-1A_, *aac*(3)*-IIa*, *sul1*, *sul2*, *dfrA1*, and *tet*(A) genes conferring resistances to penicillins, sulfonamides-trimethoprim and tetracyclines.

Finally, *mcr-1* was found on the chromosome in 32.2% (*n* = 48) of the isolates originating from three breeding and two fattening farms. Even though *mcr-1* is usually described as plasmid-borne, this proportion is very close to what has been described in healthy residents in Vietnam (36.8%; Yamaguchi et al., 2020). The presence of a unique *mcr-1*-carrying ST was observed in breeding farms #6 and #52, and in fattening farms #32 (first time point) and #55 (first and third time points). Nevertheless, chromosomal insertions of the *mcr-1* gene were also identified in different genetic backgrounds in breeding farm #19 and fattening farms #32 and #55 (both on the second time point). Among the nine isolates that were long-read sequenced, seven carried the IS*Apl1*-*mcr-1*-*pap2*-IS*Apl1* prototypic Tn*6330* transposon, which is considered as the principal vehicle of *mcr-1* spread, inserted in diverse locations on the *E. coli* chromosome. Several studies suggested that IS*Apl1* enables efficient *mcr-1* transposition through the generation of a circular intermediate (Li et al., 2018; He et al., 2019), so that transposon-mediated excision/insertion might be as successful as plasmid transfer. This composite transposon might also lose one or both IS*Apl1* sequences: this was the case in our two last sequenced isolates, which displayed a truncated chromosomally-encoded IS*Apl1*-*mcr-1*-IS*1*-like element. This was also the case in the IncHI2 and IncX4plasmid sequenced in this study, which, respectively, presented either the truncated IS*Apl1*-*mcr-1* element or only the *mcr-1*-*pap2* genes with no trace of the original IS*Apl1* sequences. Our results reinforce the hypothesis that the loss of the IS*Apl1* sequences might favor the stabilization of the *mcr-1* in a plasmidic backbone (Strepis et al., 2021).

## 5. Conclusion

Our results show that the *mcr-1* gene is actively circulating in goat production, a sector that is often overlooked. The low proportion of *mcr-1*-positive *E. coli* in goat breeding farms observed in this study (4.2% for both adults and kids; 7.4% for goat kids only) appeared sufficient to maintain this resistance gene at a high level (60.0%) in fattening farms. Only strict hygiene and biosecurity procedures in breeding farms, as well as a prudent use of antibiotics in fattening farms, can avoid such a contamination. Between- and within-farm spread of the *mcr-1* gene was also strongly suggested. This spread was due for one third each to the transmission of the IncX4 and IncHI2 plasmids as well as to the Tn*6330* transposition in diverse locations of the *E. coli* chromosome, highlighting the fact that chromosomal insertion does not impair the transmission capability of the *mcr-1* gene.

## Data availability statement

The datasets presented in this study can be found in online repositories. The names of the repository/repositories and accession number(s) can be found in the article/Supplementary material.

## Ethics statement

Written informed consent was obtained from the owners of the animal(s) for the participation in the study.

## Author contributions

MT and MH obtained the EcoAntibio2 grant. MT, J-YM, and MH designed the study. MT conducted the sampling and bacterial isolation. PC and MT performed the bench work. AD performed the WGS analyses. MH drafted the manuscript. All authors contributed to the article and approved the submitted version.

## Funding

This work was supported by internal funding of the French Agency for Food, Environmental and Occupational Health & Safety (ANSES) and by the National EcoAntibio2 Action Plan funded by the Ministry in charge of Agriculture (grant number 2017-389).

## Conflict of interest

The authors declare that the research was conducted in the absence of any commercial or financial relationships that could be construed as a potential conflict of interest.

## Publisher’s note

All claims expressed in this article are solely those of the authors and do not necessarily represent those of their affiliated organizations, or those of the publisher, the editors and the reviewers. Any product that may be evaluated in this article, or claim that may be made by its manufacturer, is not guaranteed or endorsed by the publisher.

## Footnotes

^1^ www.sfm-microbiologie.org

^2^ https://github.com/tseemann/abricate

^3^ http://www.genomicepidemiology.org/

^4^ https://github.com/tseemann/snp-dists

^5^ http://itol.embl.de/itol.cgi

^6^ http://www.genomicepidemiology.org/

^7^ https://www.bv-brc.org/
